# Racial and ethnic disparities in social isolation and 11-year dementia risk among older adults in the United States

**DOI:** 10.1017/S204579602400060X

**Published:** 2024-10-25

**Authors:** J. Grullon, D. Soong, R. Wong

**Affiliations:** 1 Norton College of Medicine, SUNY Upstate Medical University, Syracuse, NY, USA; 2 Department of Public Health and Preventive Medicine, Norton College of Medicine, SUNY Upstate Medical University, Syracuse, NY, USA; 3 Department of Geriatrics, SUNY Upstate Medical University, Syracuse, NY, USA

**Keywords:** cognitive impairment, dementia, elderly, health disparities, minority issues and cross cultural psychiatry, social environment, social network

## Abstract

**Aims:**

Social isolation has been implicated in the development of cognitive impairment, but research on this association remains limited among racial-ethnic minoritized populations. Our study examined the interplay between social isolation, race–ethnicity and dementia.

**Methods:**

We analyzed 11 years (2011–2021) of National Health and Aging Trends Study (NHATS) data, a prospective nationally representative cohort of U.S. Medicare beneficiaries aged 65 years and older. Dementia status was determined using a validated NHATS algorithm. We constructed a longitudinal score using a validated social isolation variable for our sample of 6,155 community-dwelling respondents. Cox regression determined how the interaction between social isolation and race–ethnicity was associated with incident dementia risk.

**Results:**

Average longitudinal frequency of social isolation was higher among older Black (27.6%), Hispanic (26.6%) and Asian (21.0%) respondents than non-Hispanic White (19.1%) adults during the 11-year period (*t* = −7.35, *p* < .001). While a higher frequency of social isolation was significantly associated with an increased (approximately 47%) dementia risk after adjusting for sociodemographic covariates (adjusted hazard ratio [aHR] = 1.47, 95% CI [1.15, 1.88], *p* < .01), this association was not significant after adjusting for health covariates (aHR = 1.21, 95% CI [0.96, 1.54], *p* = .11). Race–ethnicity was not a significant moderator in the association between social isolation and dementia.

**Conclusions:**

Older adults from racial-ethnic minoritized populations experienced a higher longitudinal frequency of social isolation. However, race–ethnicity did not moderate the positive association observed between social isolation and dementia. Future research is needed to investigate the underlying mechanisms contributing to racial-ethnic disparities in social isolation and to develop targeted interventions to mitigate the associated dementia risk.

## Introduction

The continuous improvement in life expectancy, among other factors, has rapidly expanded the aging population (Martinez *et al.*, 2021). In 2020, the proportion of people aged 65 years and older accounted for 9.3% of the global population, and this number is projected to reach 15.9% by 2050 (Gu *et al.*, 2021). This demographic shift presents a formidable healthcare challenge due to the increasing prevalence of age-related diseases affecting various organ systems (Chang *et al.*, 2019). Consequently, the World Health Organization’s Global Strategy and Action Plan for Aging and Health for 2016–2020 highlights the necessity of healthy aging by maintaining functional ability as life expectancy increases (Rudnicka *et al.*, 2020). This strategy focuses on addressing the physiological and psychosocial aspects of aging, such as loneliness (a subjective negative feeling with a perceived lack of social connections) and social isolation (an objective state of reduced social interactions), which are recognized as major determinants of mental health and cognitive function in older adults (Barbosa Neves *et al.*, 2019).

Earlier studies have demonstrated that indices of poor social engagement, such as a deficient social network and support, are linked to an increased dementia risk (Gardener *et al.*, 2021; Penninkilampi *et al.*, 2018), with the frequency and quality of social interactions emerging as crucial factors (Shafighi *et al.*, 2023). In recent years, the mechanism linking loneliness, social isolation and dementia risk has become increasingly apparent. For instance, neurobiological factors, such as chronic stress-related changes in brain physiology and psychosocial factors of feeling disconnected from others, may play a triggering role in the development or progression of dementia (Avila-Villanueva *et al.*, 2020). Additionally, social isolation may contribute to dementia risk by reducing hippocampal volumes and cortical thickness (Lammer *et al.*, 2023).

Interestingly, a recent study that involved two large cohorts (the UK Biobank and the Canadian Longitudinal Study of Aging) showed that loneliness and social isolation were associated with classical dementia risk across lifestyle (smoking status, alcohol intake, sleep disturbance and low physical activity), physical health (cardiovascular diseases, diabetes and vision or hearing impairment), personality traits and mental health (higher neuroticism score, depressive traits and anxiety) and external factors (social interactions, size of households or friend circle, income, occupation, education and living in urban areas) (Shafighi *et al.*, 2023). Thus, it is likely that, in addition to changes in brain physiology, loneliness and social isolation may introduce or potentiate the canonical dementia risk factors, resulting in a more rapid cognitive decline and incident dementia.

Several studies have also noted disproportionately higher rates of dementia risk factors among racial and ethnic minority groups, including but not limited to smoking, alcohol intake, sleep disturbance, physical activity, cardiovascular diseases, diabetes, depression, anxiety and lower socioeconomic status (Ahmed and Conway, 2020; Grandner *et al.*, 2016; Jehan *et al.*, 2018; Letang *et al.*, 2021; Redmond *et al.*, 2011; Zahodne *et al.*, 2017). In addition, the COVID-19 pandemic has significantly magnified social isolation issues, particularly among older age groups, with safety measures during the pandemic notably increasing social isolation, disproportionately impacting older U.S. Black and Hispanic adults (Garcia *et al.*, 2021; Millett *et al.*, 2020; Smith *et al.*, 2020). Given this agglomeration of ethnoracial disparities across adverse health behaviours, physical health, mental health and external domains, it is plausible that race and ethnicity may interact with social isolation to influence dementia risk. However, there are several gaps in our understanding of the intersectionality between social isolation, race and ethnicity, and dementia risk. For instance, there is a paucity of studies assessing longitudinal trends in social isolation and dementia risk stratified by racial and ethnic groups.

In addition, previous studies have noted ethnoracial disparities in perceived isolation and social disconnectedness, albeit with some variability depending on the data source and years analyzed (Kannan and Veazie, 2023). A secondary analysis of the 2003–2020 American Time Use Survey involving participants aged 15 years and older revealed that Black Americans spent more time in social isolation compared with other races or ethnic groups (359 hours/year more than White Americans, 663 hours/year more than Hispanic Americans and 444 hours/year more than other-race Americans) (Kannan and Veazie, 2023). Additionally, Black Americans spent less time in social engagements within the family household than other racial and ethnic groups. However, social engagement in other domains (non-household family, friends, companionship and others) was comparable between groups (Kannan and Veazie, 2023). Hispanic Americans spent the least amount of time socially isolated and the greatest amount of time socially engaged with family members (Kannan and Veazie, 2023). In contrast, a secondary analysis of the 2011 National Health and Aging Trends Study (NHATS) data indicated that both Black and Hispanic subpopulations had lower adjusted odds of social isolation than older White adults (Cudjoe *et al.*, 2022). Notably, differences in the structure, size and quality of social interactions across racial and ethnic groups are known to have significant implications for mental health, cognitive function and dementia risk (Desai *et al.*, 2020; Katz *et al.*, 2020).

Moreover, there is a pivotal gap in the application of validated measures of social isolation with psychometric reliability that resonate with the varied experiences of different racial and ethnic groups (Taylor *et al.*, 2023). Addressing this gap is imperative, as the use of non-validated or culturally biased measures can lead to inaccurate and variable estimations of the impact social isolation has on dementia risk (Donovan *et al.*, 2017; Gardener *et al.*, 2021; Huang *et al.*, 2023; Shen *et al.*, 2022). A more accurate understanding of these associations may help formulate or refine current risk assessment tools and enable greater scientific reproducibility (Guarnera *et al.*, 2023).

Therefore, this study sought to investigate (1) the association between social isolation and dementia risk, (2) the longitudinal frequency of social isolation and its variation by race and ethnicity and (3) the moderating role of race and ethnicity on the relationship between social isolation and dementia risk among older adults. Given the mixed evidence on racial and ethnic disparities in social isolation, our investigation on differences in these longitudinal trends was exploratory, however, we did hypothesize social isolation to increase dementia risk, consistent with prior research. Further, we hypothesized the association between social isolation and dementia would be moderated by race and ethnicity, due to inherent cultural differences across these groups tied to their social networks. Considering the evidence identifying social isolation as a significant risk factor for cognitive impairment, our study is poised to bridge gaps in our understanding, specifically by applying validated social isolation measures to diverse racial and ethnic groups while exploring its longitudinal impact on dementia risk. Our study seeks to harness a robust methodology to delve into the complex relationship between social isolation and dementia risk across different racial and ethnic populations, leveraging insights from other researchers in the field (Huang *et al.*, 2023; Katz *et al.*, 2020; Sutin *et al.*, 2020).

## Methods

### Data source

**Figure 1.**
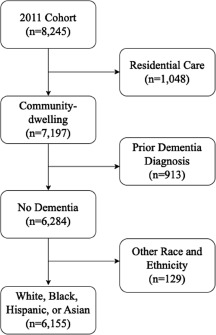
Sample selection flowchart.

Data were retrieved from the NHATS, a prospective longitudinal cohort of a nationally representative sample of U.S. Medicare beneficiaries aged 65 and older. The NHATS cohort profile and study protocol have been previously described in detail (Freedman and Kasper, 2019). The current study used data from 11 annual NHATS waves between 2011 and 2021. We restricted our sample to the initial 2011 cohort and our final sample size was 6,155 respondents who were community-dwelling, did not have dementia at baseline and self-identified as one of the racial and ethnic groups of interest (Fig. 1). Only the dementia and social isolation data were retrieved from the remaining 10 waves (2012–2021) for analyses.

### Dementia status

After removing respondents with pre-existing dementia at baseline (2011), incident dementia (2012–2021) was the dependent variable in this study. Dementia status was derived from an NHATS algorithm (Kasper *et al.*, 2013) that uses three cognitive measures: (1) the AD8 Dementia Screening Interview; (2) cognitive tests evaluating the respondents’ memory (e.g. immediate 10-word recall), orientation (e.g. date) and executive function (e.g. clock drawing test); and (3) self-report of an Alzheimer’s disease or dementia diagnosis by a doctor. From these cognitive measures, the NHATS algorithm produced a binary variable for dementia as either yes (probable dementia) or no (no or possible dementia). This narrow definition for dementia was tested to have a reasonable sensitivity of 65.7% and a high specificity of 87.2% in a consensus expert panel from the Aging, Demographics, and Memory Study (ADAMS) (Kasper *et al.*, 2013).

### Social isolation

Prior research has incorporated the Berkman–Syme Social Network Index (SNI) to create an NHATS-equivalent social isolation variable with notable convergent and divergent validity (Pohl *et al.*, 2017). To create this NHATS-equivalent social isolation variable, we followed previous guidance (Pohl *et al.*, 2017) by utilizing all 11 waves (2011–2021) of the NHATS sample person (SP) and other person (OP) files. This social isolation measure comprised of six items, with the absence of four or more of the following, indicating social isolation: (1) marriage or partner; (2) family to talk about important things; (3) friend to talk about important things; (4) visiting friends or family; (5) attending religious service; and (6) participating in clubs or organized activities. More information about the specific questions and indicators are detailed in Table 1.

**Table 1.** Social isolation variable composition

Construct	Data source	Question	Indicator^a^	
Marriage or partner	2011−2021 Sample person	“Are you currently married, living with a partner, separated, divorced, widowed, or never married?”	Separated, Divorced, Widowed, Never Married	Married, Living with a partner
Family to talk about important things	2011−2021 Other person	“Looking back over the last year, who are the people you talked with most often about important things?”	No	Yes
Friend to talk about important things			No	Yes
Visting friends or family	2011−2021 Sample person	“In the last month, did you ever visit in person with friends or family not living with you, either at your home or theirs?”	No	Yes
Attending religious service	2011−2021 Sample person	“In the last month, did you ever attend religious services?”	No	Yes
Participating in clubs or organization activities	2011−2021 Sample person	“In the last month, besides religious services, did you ever participate in clubs, classes, or other organized activities?”	No	Yes

a Absence of four or more of the six constructs reaches the threshold of social isolation based on previous psychometric testing (Pohl *et al.*, 2017).

To create longitudinal social isolation measures, we replicated an approach previously used in a study that used longitudinal NHATS data (Wong and Lovier, 2023). Binary social isolation variables from each wave were combined into a longitudinal score, which measures the proportion of years with social isolation before dementia or censoring. These longitudinal social isolation scores can range from 0 to 1 (or 0%–100%). For example, a respondent who entered the study in 2011 and died in 2016 may have reported social isolation at four annual waves during the 5-year window they were alive (from 2011 to 2015), resulting in a longitudinal social isolation score of 4/5 or 80%. Therefore, this composite score precedes dementia and represents a longitudinal measure for social isolation between 2011 and 2021, or earlier depending on when respondents were diagnosed with dementia or were censored.

### Race and ethnicity

Self-reported race and ethnicity included non-Hispanic White (hereafter, White), non-Hispanic Black (hereafter, Black), Hispanic and non-Hispanic Asian (hereafter, Asian).

### Covariates

Regression models were adjusted for sociodemographic and health variables from baseline in 2011. Sociodemographic variables included age, sex (male or female), highest level of education (less than high school, high school or college), total income and metropolitan residence (metro or non-metro). Health variables included self-rated health (poor to excellent), body mass index, limitations for activities of daily living (no ADL limitations, or at least one ADL limitation), proxy respondent, major depressive disorder (measured using Patient Health Questionnaire-2), generalized anxiety disorder (measured using Generalized Anxiety Disorder-2), heart attack history, hypertension history, diabetes history and stroke history.

### Analysis

Descriptive statistics for continuous variables were reported as mean and standard deviation, whereas categorical variables were reported as frequencies and proportions. Racial and ethnic differences in average social isolation frequency during the study period were analyzed using one-way ANOVA tests. We used Cox proportional hazards models to analyze the time (number of years) to dementia starting from baseline (2011). For our moderation analysis, we regressed dementia on the interaction between social isolation and each racial and ethnic group. To maximize the full sample size and minimize bias due to missing data (8%), multiple imputations by chained equations generated 100 imputed data files, each with 10 iterations. As recommended by the NHATS administrators, all regression models applied the round one analytic survey sampling weights with the Stata *subpop* command (Montaquila *et al.*, 2012) and were adjusted for sociodemographic and health covariates. Statistical analyses were performed in Stata statistical software version 18 (StataCorp LLC, College Station, TX, USA) with two-tailed tests at a significance level of .05.

## Results

### Sample characteristics

After applying survey sampling weights, the 6,155 NHATS respondents represented 29,736,058 older adults from the U.S. population, with a collective mean age of 74.3 years. Approximately 83.4% (*n* = 25.0 million) of respondents self-identified as White, 7.9% (*n* = 2.4 million) as Black, 6.4% (*n* = 1.9 million) as Hispanic and 2.3% (*n* = 0.7 million) as Asian. About 14.2% of respondents had an incident dementia over the 11-year study period. Respondents reported a yearly income of about $62,800 on average. Most of the respondents were female (55.4%), college-educated (63.9%), married (60.7%) and resided in a metropolitan area (81.6%).

In terms of health, most respondents self-reported to be in good health, which is supported by the observation that only some respondents had a history of depression (11.9%), anxiety (10.4%), heart attack (13.0%), diabetes (22.9%) and stroke (10.1%). Few respondents had ADL limitations (8.7%) or used a proxy respondent (1.6%). However, most respondents did have a history of hypertension (63.7%) and were overweight, with an average BMI of 27.8 kg/m^2^.

### Racial and ethnic disparities in social isolation

Respondents were, on average, socially isolated for 20.3% of the time they participated in the study from 2011 to 2021 (Table 2). There were significant differences in the average longitudinal frequency of social isolation across the four racial and ethnic groups (*t* = − 7.35, *p* < .001), with older adults of colour experiencing social isolation for a higher percentage of time during their participation. Specifically, older Asian (21.0%), Hispanic (26.6%) and Black (27.6%) adults experienced more frequent social isolation on average compared to their White counterparts (20.3%).

**Table 2.** Weighted sample characteristics^a^

Variables	Whole sample (*n* = 30.0)	White (*n* = 25.0, 83.4%)	Black (*n* = 2.4, 7.9%)	Hispanic (*n* = 1.9, 6.4%)	Asian (*n* = 0.7, 2.3%)	Bivariate test^b^
Average social isolation^c^	20.3%	19.1%	27.6%	26.6%	21.0%	*t* = −7.35, <.001
Dementia	14.2%	12.9%	19.3%	23.1%	16.2%	*F* = 10.07, <.001
Age (range 65−105)	74.3 (6.7)	74.4 (6.3)	73.4 (10.4)	73.4 (6.1)	74.0 (5.2)	*t* = 6.41, <.001
Female (%, *n*)	55.4 (16.0)	55.1 (14.0)	59.4 (1.4)	55.1 (1.0)	54.7 (0.4)	*F* = 0.88, .44
Highest Education Level (%, *n*)						
<High school	9.2 (2.7)	7.2 (1.8)	18.7 (0.4)	24.4 (0.5)	7.6 (0.05)	*F* = 37.91, <.001
High school	26.9 (8.0)	28.7 (7.1)	22.5 (0.5)	14.9 (0.3)	10.9 (0.07)	
College	63.9 (19.0)	64.1 (16.0)	58.9 (1.4)	60.7 (1.2)	81.5 (0.6)	
Income (thousands)	62.8 (196.1)	67.7 (181.8)	34.6 (113.7)	41.7 (278.0)	42.5 (45.5)	*t* = 8.42, <.001
Married (%, *n*)	60.7 (18.0)	62.6 (16.0)	40.6 (1.0)	57.1 (1.1)	69.8 (0.5)	*F* = 17.82, <.001
Metropolitan residence (%, *n*)	81.6 (24.0)	79.6 (20.0)	90.2 (2.1)	91.9 (1.7)	98.9 (0.7)	*F* = 3.13, .05
Self-rated health (0−4; Poor–Excellent)	2.4 (1.1)	2.5 (1.0)	1.9 (1.7)	1.8 (1.0)	2.1 (0.9)	*t* = 12.98, <.001
Body mass index	27.8 (5.5)	27.7 (5.0)	29.6 (10.5)	28.3 (5.0)	24.5 (3.8)	*t* = − 10.23, <.001
ADL limitations (%, *n*)	91.6 (0.6)	7.8 (1.9)	11.4 (0.3)	17.8 (0.3)	8.4 (0.1)	*F* = 16.77, <.001
No proxy respondent (%, *n*)	98.4 (29.0)	98.9 (25.0)	98.5 (2.3)	97.0 (1.8)	85.0 (0.6)	*F* = 56.13, <.001
Depression (%, *n*)	11.9 (3.5)	10.8 (2.7)	17.4 (0.4)	21.6 (0.4)	8.4 (0.1)	*F* = 12.89, <.001
Anxiety (%, *n*)	10.4 (3.1)	9.7 (2.4)	11.8 (0.3)	18.6 (0.4)	8.5 (0.1)	*F* = 10.65, <.001
Heart attack history (%, *n*)	13.0 (3.9)	13.4 (3.3)	12.0 (0.3)	10.6 (0.2)	10.6 (0.1)	*F* = 1.43, .24
Hypertension history (%, *n*)	63.7 (19.0)	61.8 (15.0)	79.7 (1.9)	66.4 (1.3)	69.7 (0.5)	*F* = 22.13, <.001
Diabetes history (%, *n*)	22.9 (6.8)	20.8 (5.2)	37.8 (0.9)	32.3 (0.6)	22.7 (0.2)	*F* = 28.39, <.001
Stroke history (%, *n*)	10.1 (0.2)	6.8 (0.1)	7.7 (0.1)	8.3 (2.5)	8.2 (2.0)	*F* = 1.20, .31

Abbreviation: ADL = activities of daily living.

a Unless otherwise indicated, data are expressed as mean (SD). All frequencies are in millions.

b ANOVA for continuous variables and Pearson chi-square test for categorical variables.

c This longitudinal score can have a range from 0% to 100%, which measures the proportion of years with social isolation prior to dementia or being censored.

**Figure 2.**
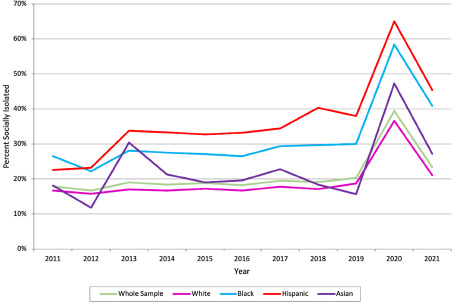
Trends in social isolation stratified by race and ethnicity.

Likewise, when examining social isolation trends cross-sectionally each year between 2011 and 2021, older adults of colour consistently experienced social isolation more often (Fig. 2). Among these groups, older Hispanic adults appeared to be the most socially isolated each year except for 2011, followed by older Black adults. Older Asian adults trended around the sample average but generally experienced slightly higher levels of social isolation. Notably, social isolation showed a slight upward trend, with a prominent spike in 2020 due to the COVID-19 pandemic, and its occurrence remained above average in 2021.

### Cox regression results

Our unadjusted crude model showed that social isolation in older adults was significantly associated with a 139% increased dementia risk (hazard ratio = 2.39, 95% CI [1.92, 2.98], *p* < .001) (Table 3, Model A). After adjusting for sociodemographics, the hazard ratio for social isolation maintained significance for a 47% increased risk of developing dementia for older adults (adjusted hazard ratio [aHR] = 1.47, 95% CI [1.15, 1.88], *p* < .01) (Table 3, Model B). However, in our fully adjusted model that accounted for co-existing health conditions, social isolation was no longer significantly associated with dementia risk (aHR = 1.21, 95% CI [0.96, 1.54], *p* = .11) (Table 3, Model C). Our interaction model did not show a significant association of race and ethnicity in moderating the relationship between social isolation and dementia risk (Table 3, Model D).

**Table 3.** Weighted Cox regression of the interaction between social isolation and race–ethnicity on dementia risk

	Hazard ratio (95%CI), *p*			
Variables	Model A	Model B	Model C	Model D
Social isolation	2.39 (1.92, 2.98), <.001	1.47 (1.15, 1.88), <.01	1.21 (0.96, 1.54), .11	1.26 (0.96, 1.65), .10
Race and Ethnicity				
White, non-Hispanic	–	Reference	Reference	Reference
Black, non-Hispanic	–	1.62 (1.35, 1.95),.001	1.47 (1.22, 1.77), < .001	1.48 (1.15, 1.91), <.01
Hispanic	–	2.04 (1.48, 2.82), <.001	1.56 (1.06, 2.30), .03	1.80 (1.17, 2.76), <.01
Asian, non-Hispanic	–	1.85 (1.09, 3.14), .02	1.49 (0.88, 2.51), .14	1.19 (0.64, 2.24), .58
Age	–	1.10 (1.09, 1.11), <.001	1.10 (1.09, 1.11), <.001	1.10 (1.09, 1.11), <.001
Female	–	1.03 (0.90, 1.17), .68	1.01 (0.89, 1.14), .90	1.01 (0.89, 1.14), .91
Highest Level of Education				
<High school	–	Reference	Reference	Reference
High school	–	0.66 (0.56, 0.77), <.001	0.73 (0.62, 0.86), <.001	0.72 (0.62, 0.85), <.001
College	–	0.62 (0.52, 0.75), <.001	0.69 (0.58, 0.83), <.001	0.69 (0.58, 0.82),.001
Income (log)	–	0.93 (0.90, 0.97), <.001	0.94 (0.90, 0.98), <.01	0.94 (0.91, 0.98), <.01
Metropolitan residence	–	0.80 (0.66, 0.96), .02	0.85 (0.72, 1.00), .05	0.85 (0.72, 1.00), .04
Self-rated health (0−4; Poor-Excellent)	–	–	0.87 (0.80, 0.94), <.01	0.86 (0.80, 0.94), <.001
Body mass index	–	–	0.98 (0.97, 1.00), .02	0.98 (0.97, 1.00), .02
ADL limitations	–	–	1.62 (1.34, 1.95), < .001	1.61 (1.33, 1.94), < .001
Proxy respondent	–	–	1.97 (1.17, 3.31), .01	2.04 (1.19, 3.49), .01
Depression	–	–	1.48 (1.21, 1.81), <.001	1.49 (1.22, 1.83), <.001
Anxiety	–	–	1.29 (1.07, 1.56), <.01	1.30 (1.08, 1.57), <.01
Heart attack history	–	–	1.15 (0.95, 1.39), .14	1.14 (0.94, 1.38), .17
Hypertension history	–	–	0.91 (0.76, 1.09), .32	0.91 (0.76, 1.10), .32
Diabetes history	–	–	1.14 (0.97, 1.34), .11	1.14 (0.97, 1.34), .10
Stroke history	–	–	1.36 (1.13, 1.64), <.01	1.35 (1.12, 1.63), <.01
Social isolation interaction				
White, non-Hispanic	–	–	–	Reference
Black, non-Hispanic	–	–	–	0.95 (0.63, 1.45), .82
Hispanic	–	–	–	0.66 (0.28, 1.55), .33
Asian, non-Hispanic	–	–	–	2.25 (0.75, 6.71), .14
Model significance	*F*(1,52) = 63.5, *p* < .001	*F*(10,54) = 84.3, *p* < .001	*F*(20,54) = 68.1, *p* < .001	*F*(23,54) = 63.6, *p* < .001

## Discussion

Our study showed that social isolation is significantly associated with an elevated dementia risk after adjusting for sociodemographic variables, but lost significance after controlling for health conditions. These findings partially agree with previous research (Evans *et al.*, 2018; Huang *et al.*, 2023). Evans *et al.* (2018) reported a significant association between baseline social isolation and cognitive change over a 2-year period in models adjusted for sociodemographic variables (age, gender and education) and physically limiting health conditions (poor eyesight and hearing or help with daily activities). These results conflicting with ours may be due to differences in how we constructed our social isolation variable along with a larger set of health variables we adjusted for in our regression model. Nonetheless, these findings confirm that social isolation should not be considered independent of overall health (Freedman and Nicolle, 2020). Instead, it is intertwined with various health factors, and accounting for co-existing health conditions is essential to understanding the relationship between social isolation and dementia risk fully. Given that social isolation was no longer statistically significant in the fully adjusted model with health covariates, it would also be worthwhile for future research to explore whether differences in health conditions may contribute to racial and ethnic disparities in social isolation.

In another analysis of the NHATS data, Huang *et al.* (2023) reported that social isolation at baseline was associated with a 33% increased risk of developing dementia during a 9-year study period (2011–2020) after adjusting for demographic factors (age, sex, race, ethnicity and education). The risk remained significant after further adjusting for health factors (number of chronic conditions, body mass index and current smoking status). However, consistent with our findings, race and ethnicity did not moderate the relationship between social isolation and dementia risk, even when a different social isolation variable was used (Huang *et al.*, 2023). Additionally, our study examined social isolation during an 11-year study period (2011–2021), and our research provides further evidence that older adults with a higher frequency of social isolation may have an increased risk of dementia.

In contrast, although not on social isolation specifically, a separate study utilizing a nationally representative sample of U.S. adults over the age of 50 examined whether race and ethnicity significantly moderated the association between social engagement through volunteering and cognitive impairment (Wang *et al.*, 2022). Their study found a decrease in the relative risk of cognitive impairment among White older adults but an increase in risk among non-Hispanic Black older adults, with every additional year engaged in informal volunteering.

Our second aim indicated older adults of colour had higher longitudinal frequencies of social isolation during the 11-year period. There are, however, inconsistent findings in previous research examining racial and ethnic disparities in social isolation. For example, in a subsample of adults aged 55 years and older from the National Survey of American Life, non-Hispanic White adults within religious congregations had greater odds of experiencing isolation and being childless than Black Americans (Taylor *et al.*, 2019). However, no significant disparities between the two groups were observed for other indicators of social isolation, such as no contact with neighbours, neighbourhood groups, family members, friends, religious congregation members, living alone, not being a parent or marital status/romantic partnerships (Taylor *et al.*, 2019). Furthermore, an analysis of Medicare beneficiaries aged 65 and older revealed that White and Black Americans showed a similar prevalence of social isolation, at 20% (Cudjoe *et al.*, 2022). While the aforementioned studies did not provide data specifically for Hispanic adults, a recent systematic review of 17 studies found inconsistent results regarding the prevalence of social isolation among older Hispanics and Latinx compared to White adults living in the U.S. (Tibirica *et al.*, 2022).

These discrepancies highlight the need for further research to establish consistent and comprehensive insights into the associations between social isolation and diverse racial and ethnic backgrounds among U.S. older adults, especially in light of the COVID-19 pandemic, which heightened social isolation among older adults (Lazzari and Rabottini, 2022). Thus, it would be worthwhile to explore whether these associations may have differed before versus after the emergence of the COVID-19 pandemic in 2020. Furthermore, additional research is required to elucidate our observation that social isolation disparities have widened over time, particularly among older Black and Hispanic adults. Given ethnoracial differences in social network quality and quantity, this may involve further exploration to determine whether it may need to develop and validate different measurements for social isolation that are more tailored to each racial and ethnic group.

Considering the inconclusive nature of social isolation research and recognizing the potential dangers of an increasingly socially isolated society, it is imperative for policymakers to prioritize funding for research on social connection and standardize data collection metrics (Office of the Surgeon General, 2023). In addition, other researchers and the U.S. Surgeon General have highlighted the need for information campaigns to inform the public and healthcare providers about the risk of cognitive decline with social isolation and the importance of funding programs that facilitate community social engagement (Jeste *et al.*, 2016; Office of the Surgeon General, 2023). Healthcare providers should regularly screen older patients with cognitive impairment for social isolation as a vital assessment component (Freedman and Nicolle, 2020). Furthermore, healthcare providers can employ motivational interviewing skills to encourage older adults to seek more social interactions, much like they would in assisting a patient with smoking cessation (Freedman and Nicolle, 2020). Although our present study indicates social isolation may be linked to increased dementia risk, additional research is also needed to identify specific aspects of social isolation (e.g. absence of family versus absence of organized activities) that may play a larger role in dementia risk so that we may develop more targeted intervention efforts.

Our study has a few limitations that should be acknowledged. First, respondents were categorized into relatively broad racial and ethnic groups, which may overlook the potential variations in habits and culture that can influence social engagement and, consequently, dementia risk. Second, the NHATS-equivalent Berkman–Syme SNI variable used in our study may be outdated and, thus, may have biased the results (Office of the Surgeon General, 2023). For instance, one of the SNI variable data points, religious attendance, has declined recently (Public Religion Research Institute, 2023). However, this trend may not necessarily translate to greater social isolation levels. Third, we could not include measures of loneliness due to survey limitations, as NHATS only incorporated survey items that measured loneliness during 2020 in the context of the COVID-19 pandemic. Finally, there is some evidence for the dual role of social isolation as a potential risk factor and a consequence of dementia, as a result of which social isolation progressively catalyzes cognitive decline (Wang *et al.*, 2022). However, our analysis specifically assessed the risk of incident dementia with social isolation. Despite these limitations, our study comprehensively explored the potential intersection between social isolation, race and ethnicity, and dementia risk using a nationally representative sample of older adults in the U.S.

## Conclusions

This study sheds light on the racial and ethnic disparities that occur due to social isolation. It underscores the potential risks of dementia in older adults, as evidenced by a nationally representative U.S. older adult sample. Moreover, we found that social isolation cannot be evaluated solely by a person’s overall health. Instead, the assessment of social isolation should consider contextual factors, which could be addressed as part of a comprehensive intervention strategy by public health and healthcare professionals to prevent and mitigate chronic diseases such as dementia in the older adult population. Future research is needed to fully explore social isolation disparities in the older adult population, investigate the underlying mechanisms contributing to these disparities, and develop targeted interventions to mitigate the associated risk of dementia.

## Data Availability

Data sharing is not because this study received approval to analyze sensitive data from the NHATS. Researchers interested in obtaining this data may submit a Sensitive Data Application to NHATS: https://nhats.org/researcher/data-access.
